# Prognostic Role of 2-[^18^F]FDG PET/CT Metabolic Volume Parameters in Patients Affected by Differentiated Thyroid Carcinoma with High Thyroglobulin Level, Negative ^131^I WBS and Positive 2-[^18^F]-FDG PET/CT

**DOI:** 10.3390/diagnostics11122189

**Published:** 2021-11-25

**Authors:** Domenico Albano, Francesco Dondi, Angelica Mazzoletti, Pietro Bellini, Carlo Rodella, Francesco Bertagna

**Affiliations:** 1 Nuclear Medicine, University of Brescia and ASST Spedali Civili Brescia, 25123 Brescia, Italy; f.dondi@outlook.com (F.D.); mazzolettiangelica@gmail.com (A.M.); bellini.pietro@outlook.it (P.B.); francesco.bertagna@unibs.it (F.B.); 2 Health Physics Department, ASST-Spedali Civili, 25123 Brescia, Italy; carlo.rodella@asst-spedalicivili.it

**Keywords:** DTC, thyroid cancer, 2-[^18^F]FDG PET/CT, MTV, TLG

## Abstract

The clinical and prognostic role of 2-deoxy-2-[^18^F]fluoro-D-glucose positron emission tomography/computed tomography (2-[^18^F]FDG PET/CT) in the study of patients affected by differentiated thyroid carcinoma (DTC) with positive serum thyroglobulin (Tg) level and negative [^131^I] whole-body scan ([^131^I]WBS) has already been demonstrated. However, the potential prognostic role of semi-quantitative PET metabolic volume features, such as metabolic tumor volume (MTV) and total lesion glycolysis (TLG), has not yet been clearly investigated. The aim of this retrospective study was to investigate whether the main metabolic PET/CT parameters may predict the prognosis. We retrospectively included 122 patients with a positive 2-[^18^F]FDG PET/CT for DTC disease after a negative [^131^I]WBS with Tg > 10 ng/mL. The maximum and mean standardized uptake value (SUVmax and SUVmean), MTV and TLG of the hypermetabolic lesion, total MTV (tMTV) and total TLG (tTLG) were measured for each scan. Progression-free survival (PFS) and overall survival (OS) curves were plotted according to the Kaplan–Meier analysis. After a median follow up of 53 months, relapse/progression of disease occurred in 87 patients and death in 42. The median PFS and OS were 19 months (range 1–132 months) and 46 months (range 1–145 months). tMTV and tTLG were the only independent prognostic factors for OS. No variables were significantly correlated with PFS. The best thresholds derived in our sample were 6.6 cm^3^ for MTV and 119.4 for TLG. In patients with negative WBS and Tg > 10 ng/mL, 2-[^18^F]FDG PET/CT metabolic volume parameters (tMTV and tTLG) may help to predict OS.

## 1. Introduction

Differentiated thyroid cancer (DTC) is the most frequent endocrine cancer and it is a disease with optimal survival [1], except in cases with distant metastases (mainly bone) and iodine-refractory states [2,3]. The initial therapy for DTC consists of surgery (thyroidectomy with/without lymphadenectomy) followed by postoperative risk-adapted sodium iodide ([^131^I]) therapy if indicated [1,2,3,4,5]. This approach leads to an excellent response in most cases [6,7], but there is a reduced number of cases where conventional treatments are less effective and prognosis becomes poor. The goal to identify the patients who will have a more aggressive disease would be shareable, especially in an early stage.

In this clinical scenario, the combination of the serum thyroglobulin (Tg) level and [^131^I] whole-body scan ([^131^I]WBS) findings may help to recognize patients who have not have benefited from [^131^I] therapy. In fact, in the presence of negative [^131^I]WBS and detectable Tg, iodine refractory disease is defined [8,9].

For the study of patients with negative [^131^I]WBS and positive Tg, 2-deoxy-2-[^18^F]fluoro-d-glucose positron emission tomography/computed tomography (2-[^18^F]FDG PET/CT) is suggested for a better definition of disease status and for the prognostication [10,11]. Moreover, recent papers [12,13] suggested a strong correlation between 2-[^18^F]FDG PET/CT results and Tg kinetics, expressed as Tg-velocity and/or Tg-doubling time. The potential prognostic impact of 2-[^18^F]FDG PET/CT in DTC was suggested by Wang et al. in 2000 [14]. Since then, other articles [15,16,17,18] have focused on the prognostic role of 2-[^18^F]FDG PET/CT. In these papers, PET/CT was analyzed by a qualitative point of view dichotomizing the PET/CT scans as positive or negative according to the presence of increased FDG localizations. Instead, a clear evaluation of semi-quantitative PET parameters related to the volume is not yet available; only initial promising findings in small samples of patients seem to underline a potential usefulness of these variables [19,20,21].

The aim of this retrospective study was to assess the prognostic role of PET metabolic volume parameters in patients affected by DTC with a high thyroglobulin level, negative [^131^I]WBS scan and a positive 2-[^18^F]FDG PET/CT.

## 2. Materials and Methods

### 2.1. Patients

**Figure 1:**
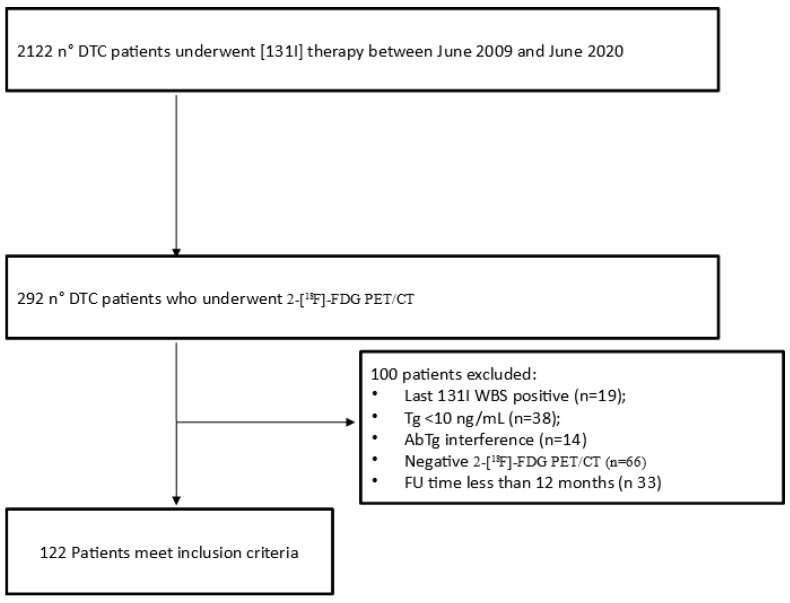
Flowchart of patients included.

From June 2009 to June 2020, 122 patients who received [^131^I] therapy for DTC after total or nearly total thyroidectomy were retrospectively screened. They were admitted to our Nuclear Medicine Unit for ablation of the thyroid remnant and/or subsequent [^131^I] therapy if indicated according to EANM (European Association of Nuclear Medicine) guidelines [22]. Among these patients, we recruited all patients who underwent a 2-[^18^F]FDG PET/CT scan during the course of the disease. Inclusion criteria were (a) the presence of a positive 2-[^18^F]FDG PET/CT scan; (b) the presence of a negative [^131^I]WBS near to the PET/CT; (c) a Tg value ≥ 10 ng/mL at the time of the PET/CT scan; (d) no detectable Tg-antibodies; (e) age higher than 18 years old at the time of PET/CT; (f) follow-up time more than 12 months from PET/CT or until death. Finally, 122 (6%) patients were investigated (Figure 1). The main epidemiological and clinical features of this sample are given in Table 1. All PET/CT scans were performed after more than one radiometabolic treatment and the mean time between the last [^131^I] therapy and PET/CT was 6 months (range 2–13 months). There was a prevalence of male (*n* = 67) compared to female (*n* = 55). Average age was 56 years (range 20–80). The classic variant of papillary carcinoma was the most frequent histotype with 46 cases (38%), followed by follicular carcinoma (20%) and aggressive papillary variants (19%) (15 tall cell variants of papillary carcinoma and 8 sclerosing diffuse variants of papillary carcinoma). The average size of the primary tumor was 35 mm (range 5–90 mm). Considering ATA class risk categories at ablation, 16 (13%) patients were in the low-risk group, 80 (66%) in the intermediate risk-group and 26 (21%) in the high-risk group. Average Tg at ablation was 236 ng/mL (0.1–1001 ng/mL), while at the time of 2-[^18^F]FDG PET/CT was 165 ng/mL (10–2020 ng/mL).

### 2.2. 2-[^18^F]-FDG PET/CT Imaging and Interpretation

All patients underwent 2-[^18^F]FDG PET/CT following the recommendations of EANM [22]. At least 6 h of fasting was requested to the patients before performing PET/CT and the glucose level at the time of radiotracer injection was required to be less than 150 mg/dL. Radiotracer activity injected was 3.5–4.5 MBq/Kg and was administered intravenously; the scan was acquired about 60 min after radiotracer injection from the vertex to the mid-thigh on a Discovery ST or Discovery 690 PET/CT tomograph (General Electric Company—GE^®^—Milwaukee, WI, USA). PET bed-time was 2.5 min; axial width was 15 cm. The reconstruction was performed in a 256 × 256 matrix with a 60 cm field of view. For both scanners a standard non-contrast free-breathing helical low-dose CT (80 mA, 120 Kv without contrast) was performed for morphological correlation and attenuation correction purposes. The acquisition parameters were 120 kV, fixed tube current 40–160 mAs, 64 slices × 3.75 mm and 3.27 mm interval, tube rotation 0.5–0.8 s. The PET images were analyzed qualitatively by a nuclear medicine physician with long experience in this field (F.B.) and were judged as positive in the presence of any focal FDG uptake higher than from the physiological background. The same researcher performed a semi-quantitative analysis by estimating several semiquantitative PET parameters, such as SUVmax (the maximum standardized uptake value) of the hypermetabolic lesion, SUVmean (the mean standardized uptake value) of the hypermetabolic lesion, metabolic tumor volume of the hypermetabolic lesion (MTV), total lesion glycolysis of the hypermetabolic lesion (TLG), total MTV (tMTV) and total TLG (tTLG). For the measurement of SUVmax and SUVmean a region of interest (ROI) of 10 mm was draw to the area of maximum activity; SUVmax and SUVmean were calculated as the highest and average SUV values of the pixels within the ROI. For the measurement of MTV an automated contouring program (Advantage Workstation 4.6, GE HealthCare) was used with an isocounter threshold method based on 41% of the SUVmax, as suggested by EANM guidelines [23]. Total MTV (tMTV) was obtained by the sum of all lesions with increased FDG uptake. For the calculation of TLG, the sum of the product of MTV of each lesion and its SUVmean was performed.

### 2.3. Thyroglobulin Evaluation

Tg was measured by an immunoradiometric assay (DYNOtest^®^ Tg-plus; BRAHMS Diagnostica, Hennigsdorf, Germany) according to the manufacturer’s instructions. The presence of autoantibodies against Tg was evaluated by a specific radioimmunoassay (DYNOtest^®^ anti-Tg_n_; BRAHMS Diagnostica, Hennigsdorf, Germany). The last Tg measurement near to the 2-[^18^F]-FDG PET/CT was selected; Tg was measured within a mean time of 5 days prior to PET/CT (range 0–25 days). In 45 cases, the Tg under thyrotropin suppression therapy was available. In the remaining 77 cases the stimulated Tg was considered: 50 times after levothyroxine withdrawal (for 40 days, replaced by triiodothyronine in the first 20 days), 27 times after recombinant human thyrotropin (rhTSH) stimulation. Recombinant human thyrotropin (rhTSH) (Genzyme Corporation, Cambridge, MA, USA) was administered intramuscularly with a dose of 0.9 mg on 2 consecutive days during treatment with levothyroxine.

### 2.4. Statistical Analysis

The statistical analysis was carried out using MedCalc Software version 17.1 for Windows (Ostend, Belgium) and/or Statistical Package for Social Science (SPSS) version 23.0 for Windows (IBM, Chicago, IL, USA). The numeric variables were presented as mean, minimum, maximum and standard deviation (SD). The categorical variables were presented as simple and relative frequencies. For the entire population, a receiver operating characteristic (ROC) curve analysis was used to identify the optimal cutoff point of semiquantitative parameters in the light of which we interpreted the results of progression-free survival (PFS) and overall survival (OS) (Table 2). OS was calculated from the date of 2-[^18^F]FDG PET/CT to the date of death from any cause or to the date of last follow-up. PFS was calculated from the date of baseline 2-[^18^F]-FDG PET/CT to the date of first disease progression, relapse, death or the date of last follow-up. PFS and OS curves were plotted according to the Kaplan–Meier method and differences between groups were analyzed by using a two-tailed log rank test. Cox regression was used to estimate the hazard ratio (HR) and its confidence interval (CI). A *p* value of <0.05 was considered statistically significant.

## 3. Results

### 3.1. 2-[^18^F]-FDG PET/CT Findings

At the time of 2-[^18^F]FDG PET/CT, the mean serum Tg was 165 ng/mL (range 10–2020 ng/mL). In 78 patients (64%) 2-[^18^F]FDG PET/CT showed FDG increased uptake corresponding to disease localizations (36 only in lungs, 7 only in skeleton, 22 in coexisting lymph node and lung and/or bone metastases, 14 in coexisting bone and lung metastases, 15 in cervical lymph nodes, 14 in thyroid tissue bed plus cervical lymph nodes, 6 only in thyroid tissue bed, 3 in coexisting lung plus bone plus liver metastases, 2 in liver, 1 in brain and lungs, 1 only in brain and only 1 in muscle). The confirmation of the PET/CT findings was obtained by a fine-needle aspiration in 15 cases (8 in thyroid bed and 9 in cervical nodes), by post-surgery histological examination in 15 cases (1 in thyroid bed, 4 in thyroid bed plus cervical nodes, 8 only in cervical nodes and 2 in lung metastases) and by a combination of clinical, biochemical and radiological evidence in the remaining cases.

**Figure 2:**
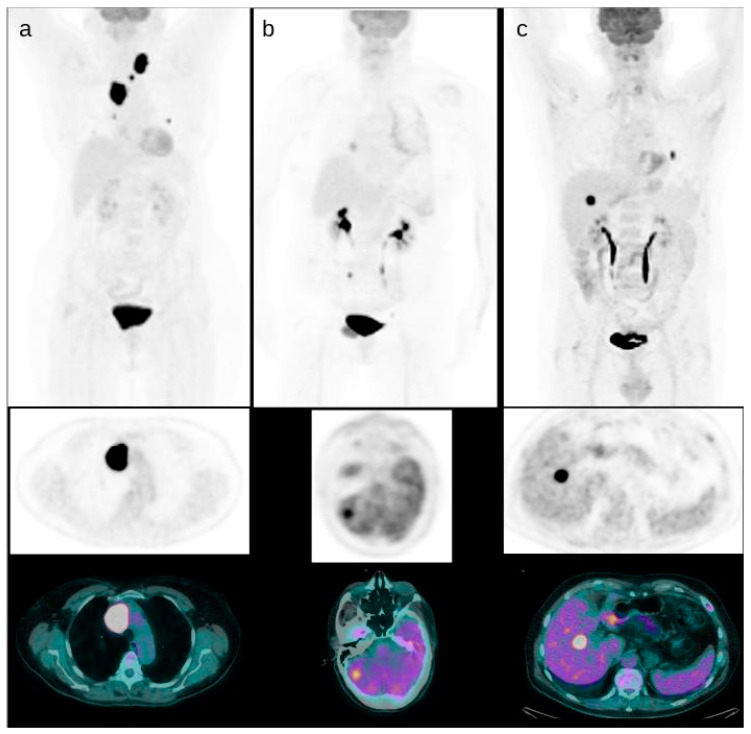
Some examples of DTC patients with positive PET/CT. A patient with mediastinal and thoracic FDG positive disease and the lesion with higher uptake in the upper mediastinum ((**a**), SUVmax 55). A patient with brain metastases detected by PET/CT with uptake higher than the surrounding brain parenchyma ((**b**), SUVmax 20). A patient with liver metastases ((**c**), SUVmax 35).

The average number of lesions for examination was 4.3 ± 4.1 (range 1–23). The average ± SD SUVmax, SUVmean, MTV and TLG of the lesion with the highest FDG uptake were 30.4 ± 29.5, 13.7 ± 13.9, 5.8 ± 5.1, 139.2 ± 152.4, respectively (Figure 2). Instead, tMTV and tTLG were 33.6 ± 16.6 and 523 ± 278. After 2-[^18^F]FDG PET/CT, 26 patients (21%) underwent a surgical intervention, 7 patients (6%) radiotherapy treatment, 8 patients (7%) systematic therapy with tyrosine kinase inhibitor (seven with Lenvatinib, 1 with Sorafenib), 1 (1%) patient received chemotherapy, 1 (1%) patient hepatic chemoembolization, 19 patients (16%) a new [^131^I] therapy and 60 (49%) continued a thyroid hormone suppression therapy without specific treatments.

### 3.2. Role of 2-[^18^F]FDG PET/CT in Predicting the PFS and OS

**Figure 3:**
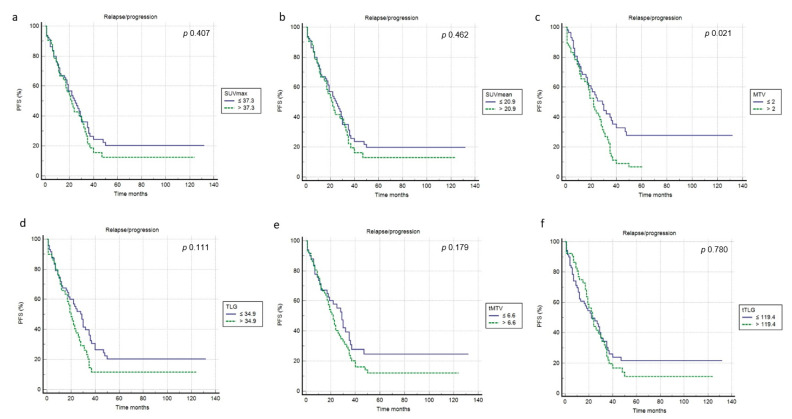
Kaplan–Meier curves relating to PFS and the PET/CT metrics ((**a**) SUVmax; (**b**) SUVmean; (**c**) MTV; (**d**) TLG; (**e**) tMTV; (**f**) tTLG).

**Figure 4:**
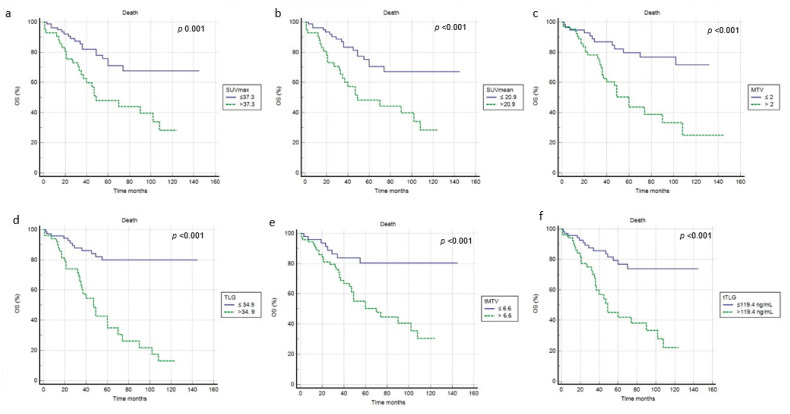
Kaplan–Meier curves relating to OS and the PET/CT metrics ((**a**) SUVmax; (**b**) SUVmean; (**c**) MTV; (**d**) TLG; (**e**) tMTV; (**f**) tTLG).

At a median follow-up of 53 months, a relapse or progression of disease occurred in 87 patients with an average time of 18 months (range: 1–50 months) from the baseline 2-[^18^F]FDG PET/CT, while death occurred in 42 patients with an average time of 35.3 months (range 1–108). The median PFS and OS were 19 months (range 1–132 months) and 46 months (range 1–145 months). Applying ROC curve analysis (Table 2), we derived the best cut-off values for SUVmax, SUVmean, MTV, TLG, tMTV and tTLG of 37.3, 20.9, 2, 34.9, 6.6 and 119.4, respectively. Considering PFS, at univariate analysis no metabolic PET parameters included were significantly correlated with survival (Figure 3, Table 3). In addition, none of the other epidemiological, clinical and histological features were associated with PFS. Considering OS, at univariate analysis all metabolic PET parameters and the presence of bone metastases were significantly related to survival curves (Figure 4, Table 3) together with Tg value at the time of PET/CT. At multivariate analysis, only the presence of bone metastases, tTLG and tMTV were confirmed to be independently correlated with OS. Patients with a high tMTV (>6.6 cm^3^) had a significantly shorter OS compared to patients with a lower tMTV (≤6.6 cm^3^), with a median OS of 39 months compared to 54 months (*p* < 0.001). Considering 57 patients with low-tMTV, 12 (21%) died, while among 65 patients with high tMTV 30 (46%) died. Considering DTC patients with high TLG (>119.4), OS was significantly shorter compared to low-TLG (≤119.4), with a median OS of 35% compared to 55% (*p* < 0.001). Of 70 patients with low-tTLG, 14 (20%) died, while 52 patients with high tTLG 28 (54%) died.

## 4. Discussion

Despite the overall optimal prognosis, a reduced group of DTC patients lost the ability to take up radioiodine and shifted into an aggressive behavior, which may usually manifest with a high level of Tg but negative or less positive [^131^I]WBS. These patients are a clinical challenge because the therapeutic options are decreased and the prognosis is worse. The main international guidelines [1,24] have recommended 2-[^18^F]FDG PET/CT as an imaging tool for studying DTC patients with aggressive disease or with elevated Tg and negative WBS during follow-up. The underlying mechanism of FDG avidity in DTC cells is related to the upregulation of glucose transporter-1 (GLUT1) and reduced expression of sodium-iodide symporter (NIS) [25]; this process is called the flip-flop phenomenon [26,27] and may explain the usefulness of 2-[^18^F]FDG PET in aggressive DTC. The worse prognosis of positive 2-[^18^F]FDG PET/CT compared to a negative PET/CT scan has already been demonstrated with solid evidence. Robbins et al. [15] analyzed more than 400 patients and demonstrated that patients with positive 2-[^18^F]FDG PET had a 7-fold increased risk of mortality in comparison with patients with negative 2-[^18^F]FDG PET. However, among positive PET/CT, it is not yet clear if any differences related to the risk of recurrence/progression or death are present. Moreover, the potential impact of PET/CT metabolic metric parameters has not been clearly investigated. SUV was the first metabolic index investigated with controversial results [19,28]. In this scenario the introduction of alternative PET/CT parameters, expressing at the same time the metabolic and volumetric characteristics of 2-[^18^F]FDG-positive lesions, showed a significant role in several solid tumors. In our study, we analyzed several PET metrics (SUVmax, SUVmean, MTV and TLG often hypermetabolic lesion, tMTV and tTLG) demonstrating that only tMTV and tTLG were independently correlated with OS at multivariate analysis. These parameters were even better than the main clinical, epidemiological and biochemical features. Instead, considering PFS, no significant associations were discovered. This discrepancy between OS and PFS may be due to the high aggressiveness of most of the patients analyzed. In fact, 87/122 patients had relapse or progression of disease after an average time of 18 months from PET/CT. Another relevant finding of our work is the prognostic superiority demonstrated by tMTV and tTLG compared to other PET/CT features (SUV and MTV and TLG of the primary lesion). The potential limitations of SUV are intrinsically derived by its definition. It may be affected by different conditions related to the patient (body weight), to the examination (uptake time, risk of extravasation, residual activity in device), to the radiotracer (decay, activities injected), to the scanner used (technology, acquisition and reconstruction protocol) and to the disease (size, localization, partial volume effect). All these factors may explain the different results present in the literature about the role of SUV in DTC restaging. Moreover, SUV is usually measured only in one lesion (that with higher 2-[^18^F]FDG uptake); thus, it does not represent the real status of disease of a patient, especially when is it multi-metastatic. For the same reason, we can justify the better performance of tMTV and tTLG in comparison with MTV and TLG of the hypermetabolic lesion. MTV and TLG may be considered a compromise between morphological and functional data, because they reflect both the volume of a lesion and its metabolic aggressiveness, expressed as 2-[^18^F]FDG uptake. However, the biggest limitation for the clinical routine applications of these parameters is the lack of methodological standardization and reproducibility. Different methods are proposed in the literature, with fixed (i.e., SUV = 2.5) or threshold (i.e., 41% of SUVmax) or combination segmentation models. Until now, no method seems to be superior to others, even if EANM guidelines [23] have suggested an isocounter threshold method based on 41% of the SUVmax. In addition, Manohar et al. [19] in a smaller population (*n* = 62) demonstrated a prognostic role of MTV and TLG both for OS and PFS. They dichotomized MTV and TLG according to the median values and then analyzed the log-MTV and log-TLG. Instead, in our paper we preferred to apply ROC curve analysis to find the point with the best compromise between sensitivity and specificity. Rizzini et al. [19] showed a positive correlation between MTV and TLG and Tg level and the final disease status, but they did not focus on the outcome survival. Surely the cutoffs derived by this work (6.6 for tMTV and 119.4 for tTLG) are not reproducible at the moment because they are strictly related to the population analyzed and the center’s features; however, they could be used as starting points for future analysis on large multicentric populations. Aside from metabolic parameters, the presence of bone metastases resulted in an independent prognostic factor for OS, as is known from the literature [1].

Bone and lungs were the most frequent sites of distant metastases in DTC, but rarer localizations are also possible [29,30]

A further potential help in the study of this kind of patients could derive from the introduction of PET/MRI instead of PET/CT.

There are several potential advantages of this tool, such as the radiation dose reduction, a better accuracy in the local tumor extent evaluation and an excellent soft-tissue contrast. A direct comparison between 2-[^18^F]FDG PET/CT and PET/MRI showed a similar accuracy between the two methods [31,32]. Nevertheless, the real usefulness of PET/MRI needs to be clarified with a larger sample of patients.

Our study presents some limitations: first, the retrospective design of the work; second, the relatively low sample of patients recruited, also due to the rarity of the condition analyzed.

## 5. Conclusions

In conclusion, with this study we have demonstrated that 2-[^18^F]FDG PET/CT metabolic tumor volume features (tMTV and tTLG) were significantly correlated with OS, but not with PFS, in DTC patients with negative radioiodine scans and detectable Tg. Metabolic tumor volumes may have an impact in recognizing patients with aggressive disease needing personalized management.

## Figures and Tables

**Table 1:** The main characteristics of our patients (*n* = 122).

	Mean SD (Range)	Patients *n* (%)
Age years	56 ±14.4 (20–80)	
Sex		
Male		67 (55%)
Female		55 (45%)
Histotype		
Papillary		46 (38%)
Follicular variant of papillary		11 (9%)
Follicular		24 (20%)
Aggressive variant		23 (19%)
Hurtle cell		17 (14%)
Unknown (Tx)		1 (1%)
Tumor size (mm)	35 ± 15 (5–90)	
Multicentricity		25 (20%)
Thyroiditis		13 (10%)
T-stage at staging		
sTx		1 (1%)
sT1		8 (7%)
sT2		22 (18%)
sT3		69 (56%)
sT4		22 (18%)
N-stage at staging		
sN0		70 (57%)
sN1a		32 (26%)
sN1b		20 (14%)
M-stage at staging		
sM1		5 (9%)
ATA class risk		
Low		16 (13%)
Intermediate		80 (66%)
High		26 (21%)
Tg at the time of ablation (ng/mL)	236 ± 962 (0.1–1001)	
TgAb positive at ablation		18 (15%)
Tg at time of PET/CT	165 ± 320 (10–2020)	
First RAI activities administrated (GBq)	3.2 ± 1 (1–5.5)	
Cumulative RAI activities administrated (GBq)	310 ± 18.2 (3.7–77.7)	
N° RAI therapies	4.4 ± 2.3 (1–10)	

**Table 2:** Threshold values derived by using ROC curve analysis considering the entire population and death as final reference.

Parameter	AUC (95% CI)	*p* Value	Cut-Off Value	Youden Index	Sensitivity	Specificity
SUVmax	0.662	0.003	37.3	0.346	57%	77.5%
SUVmean	0.652	0.006	20.9	0.346	57%	77.5%
MTV	0.662	0.002	2	0.276	71%	56%
TLG	0.719	<0.001	34.9	0.464	71%	75%
tMTV	0.702	<0.001	6.6	0.334	81%	53.5%
tTLG	0.729	<0.001	119.4	0.366	67%	70%

**Table 3:** Univariate and multivariate analyses for PFS and OS.

	Univariate Analysis		Multivariate Analysis	
	*p* Value	HR (95% CI)	*p* Value	HR (95% CI)
PFS				
Sex	0.123	1.399 (0.910–2.150)		
ATA class risk	0.158	0.662 (0.345–1.183)		
Age	0.224	0.789 (0.400–1.540)		
Tumor size	0.450	1.240 (0.778–2.234)		
Multicentricity	0.637	1.187 (0.581–2.423)		
Histotype	0.849	1.376 (0.590–3.184)		
Number of lesions at PET	0.123	1.125 (0.874–1.201)		
Presence of bone metastases	0.200	1.900 (0.881–2.001)		
Cumulative RAI activities administrated (GBq)	0.507	1.308 (0.666–1.998)		
N° radiometabolic therapies	0.562	1.284 (0.712–1.999)		
Tg at PET	0.395	1.237 (0.757–2.029)		
Watchful waiting approach	0.825	0.959 (0.620–1.462)		
SUVmax *	0.407	1.208 (0.771–1.892)		
SUVmean *	0.462	1.184 (0.754–1.858)		
MTV	0.214	1.666 (0.890–2.574)		
TLG	0.111	1.445 (0.918–2.275)		
tMTV *	0.178	1.344 (0.872–2.073)		
eTLG *	0.780	1.063 (0.690–1.633)		
**OS**				
Gender	0.390	1.513 (0.624–4.044)		
ATA class risk	0.370	0.445 (0.286–1.145)		
Age	0.450	0.888 (0.330–3.325)		
Tumor size	0.570	0.845 (0.400–2.252)		
Multicentricity	0.480	0.811 (0.319–3.325)		
Histotype	0.254	0.568 (0.233–5.561)		
Number of lesions at PET	0.530	1.358 (0.666–1.501)		
Presence of bone metastases	0.001	2.194 (1.001–4.222)	0.009	3.999 (1.987–6.980)
Cumulative RAI activities administrated (GBq)	0.219	1.046 (0.974–1.124)		
N° radiometabolic therapies	0.279	0.736 (0.413–1.298)		
Tg at PET	0.048	2.741 (0.964–7.787)	0.321	0.850 (0.650–2.001)
Watchful waiting approach	0.215	0.678 (0.369–1.251)		
SUVmax *	0.001	2.943 (1.544–5.603)	0.221	0.991 (0.229–13.054)
SUVmean *	0.007	3.053 (1.598–5.832)	0.145	2.280 (0.263–16.121)
MTV	<0.001	3.240 (1.741–6.303)	0.099	1.152 (0.881–2.354)
TLG	<0.001	6.051 (3.152–11.615)	0.062	1.122 (0.789–2.266)
tMTV *	<0.001	2.894 (1.567–5.344)	0.029	2.539 (1.097–5.874)
eTLG *	<0.001	3.799 (2.015–7.163)	0.014	2.477 (1.198–5.123)

PFS: progression-free survival; OS: overall survival; HR: hazard ratio; CI: confidence interval; N°: number; SUV: standard uptake value; MTV: metabolic tumor volume; TLG: total lesion glycolysis. * Variables dichotomized using cutoff values after ROC analysis reported in Table 2.

## Data Availability

The data presented in this study are available on request from the corresponding author.
